# Electrochemical Impedance Analysis of a PEDOT:PSS-Based Textile Energy Storage Device

**DOI:** 10.3390/ma11010048

**Published:** 2017-12-28

**Authors:** Ida Nuramdhani, Argun Talat Gokceoren, Sheilla Atieno Odhiambo, Gilbert De Mey, Carla Hertleer, Lieva Van Langenhove

**Affiliations:** 1 Department of Materials, Textiles, and Chemical Engineering, Centre for Textile Science and Engineering, Ghent University, B-9000 Gent, Belgium; carla.hertleer@gmail.com (C.H.); Lieva.VanLangenhove@UGent.be (L.V.L.); 2 Department of Textile Chemistry, Polytechnic STTT Bandung, Bandung, Jawa Barat 40272, Indonesia; 3 Science and Letters Faculty, Chemistry Department, Istanbul Technical University, Maslak, 34469 Istanbul, Turkey; gokceorena@itu.edu.tr; 4 Department of Manufacturing, Industrial & Textile Engineering, Moi University, Eldoret, 30100-Rift Valley, Kenya; sheillatienoodhiambo@gmail.com; 5 Department of Electronics and Information Systems, Faculty of Engineering and Architecture, Ghent University, B-9000 Gent, Belgium; gilbert.demey@ugent.be

**Keywords:** textile device, energy storage, PEDOT:PSS, electrochemical impedance spectroscopy

## Abstract

A textile-based energy storage device with electroactive PEDOT:PSS (poly(3,4-ethylenedioxythiophene)/poly(4-styrenesulfonate)) polymer functioning as a solid-state polyelectrolyte has been developed. The device was fabricated on textile fabric with two plies of stainless-steel electroconductive yarn as the electrodes. In this study, cyclic voltammetry and electrochemical impedance analysis were used to investigate ionic and electronic activities in the bulk of PEDOT:PSS and at its interfaces with stainless steel yarn electrodes. The complex behavior of ionic and electronic origins was observed in the interfacial region between the conductive polymer and the electrodes. The migration and diffusion of the ions involved were confirmed by the presence of the Warburg element with a phase shift of 45° (*n* = 0.5). Two different equivalent circuit models were found by simulating the model with the experimental results: (QR)(QR)(QR) for uncharged and (QR)(QR)(Q(RW)) for charged samples. The analyses also showed that the further the distance between electrodes, the lower the capacitance of the cell. The distribution of polymer on the cell surface also played important role to change the capacitance of the device. The results of this work may lead to a better understanding of the mechanism and how to improve the performance of the device.

## 1. Introduction

Research on flexible and wearable electronics (e-textiles) has been gaining momentum in recent years with a wide range of applications covering the use in medical, military, sport and outdoor activities as well as everyday consumer applications. One of the very first reports on textile batteries or supercapacitors was that of Bhattacharrya et al. [1]. The work was focused on fabrication of a simple textile energy storage device by using a coating of PEDOT:PSS as a solid electrolytic layer covering three parallel silver-coated polyamide yarn electrodes on a polyamide fabric. This device has attracted our attention and became the basis for our work on textile energy storage devices. Despite its facile design and simple fabrication, the device demonstrated a promising performance. In our previous works [2], we have shown that stainless-steel yarns gave better results than the alternative silver-coated PBO (polybenzoxazol) filament yarns. The less expensive electroconductive stainless-steel filament yarns showed higher capability to store charge in a longer time. Additionally, the stainless steel yarns also showed good electrical and thermal conductivity compared with normal yarns that gave further advantages. Thus, stainless-steel filament yarns were used as the electrodes for our textile-based energy storage device while PEDOT:PSS, presumably acted as the electrolyte, facilitated the generation of electrical energy.

One of the main issues that has attracted a considerable interest and attention in the development of textile energy devices is the underlying mechanisms of the device. There is a general and fundamental difference between the mechanisms of operation of electrochemical capacitors and battery cells [3]. Ideally, with the double-layer type of capacitor (EDLCs), there is no electron transfer across the electrode interface and the storage of electric charge and energy is electrostatic. Charge accumulation is achieved electrostatically by positive and negative charges residing on two interfaces separated by a vacuum or a dielectric. In battery-type processes, electron transfer takes place across the double layers. The charge storage is achieved by an electron transfer that produces chemical or oxidation state changes in the electroactive materials according to Faraday’s laws related to electrode potential. Compared to batteries, supercapacitors or electrochemical capacitors demonstrate higher performance than batteries especially in terms of their power density, life cycle, and charge-discharge rate [4], although some shortcoming issues such as high current leakage and low voltage window are still of concern in the field of research [5]. According to Bhattacharya et al. [1], the device they have developed worked based on the principles of a battery where a chemical interaction occurred at the Ag/PEDOT:PSS interface. SEM (Scanning electron microscopy) imaging and energy dispersive X-ray (EDX) analysis on the device have shown the movement of silver ions through the PEDOT:PSS from the anode to cathode under the bias of high electric field. However, our works with stainless-steel electrode yarns have indicated that possible other mechanism may also have come into play suggesting intermediate situations or hybrid mechanisms between battery and capacitors [2,6]. A possible mechanism of charge storage could be that of charge separation within the structure of PEDOT:PSS. Studies have shown that PEDOT:PSS undergoes conformation changes from coil to more linear conformation under the effect of electric field followed by charge separation between the positively charged PEDOT and negatively charged PSS—each attracted toward negative and positive electrode respectively [7,8,9,10,11,12,13]. Thus, it is plausible that both double layer capacitance and surface redox reaction exist in the operation of the device, hence the possibility of having a pseudocapacitor.

Electrochemical impedance spectroscopy (EIS) is a sensitive technique for determining the mechanistic pathway of an electrochemical reaction [14]. It has been one of the routine analytical tools for the characterization and diagnosis of capacitors including textile energy storage devices. It allows estimation of frequency behavior, quantification of resistance, and the ability to model equivalent circuits of capacitor systems. EIS has been used to study the mechanisms of charge storage of different materials in different electrochemical capacitors [14,15,16,17,18,19,20,21,22]. Cyclic voltammetry and constant current charge discharge methods were used in conjunction with EIS to discern different storage mechanisms which are knowingly unique for each of the materials [15].

In this paper, cyclic voltammetry and EIS were used to investigate the ionic and electronic activity within the bulk of PEDOT:PSS and at its interfaces with stainless-steel yarn electrodes in our textile energy device. Equivalent circuit models were adopted as an approach to elucidate the contribution of different charge transfer or transport processes to the overall impedance of electrodes in the device.

## 2. Results and Discussion

### 2.1. Cyclic Voltammetry

**Figure 1:**
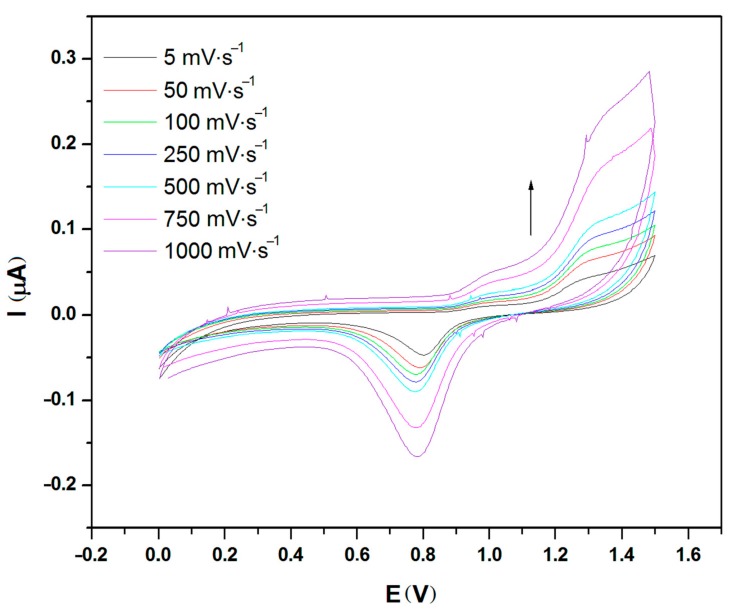
Cyclic voltammogram at various scan rate for PEDOT:PSS solution.

**Figure 2:**
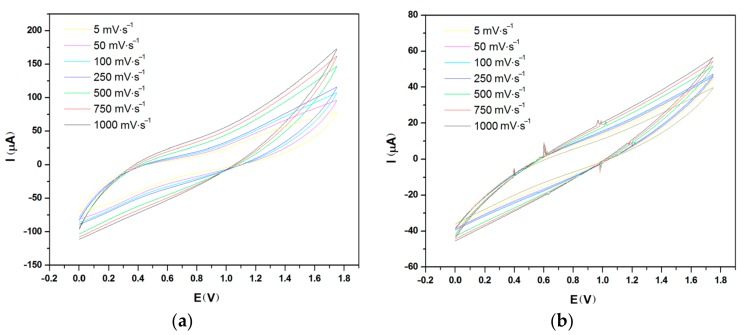
Cyclic voltammogram at various scan rate for sample on glass surface with electrode distance *d* of (**a**) 3 mm and (**b**) 6 mm.

**Figure 3:**
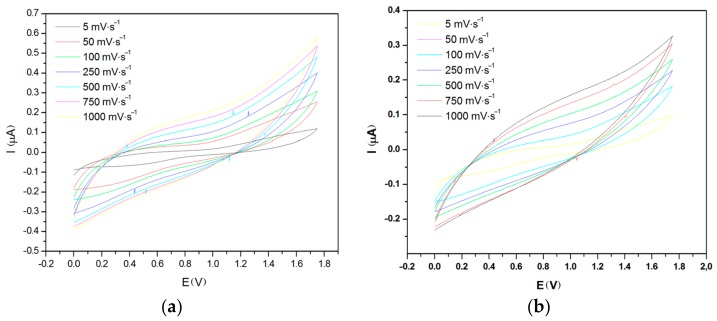
Cyclic voltammogram at various scan rate for (**a**) 3-mm and (**b**) 6-mm sample on fabric surface.

Figure 1, Figure 2 and Figure 3 show the cyclic voltammograms of PEDOT:PSS in aqueous dispersion and in the form of thin films on glass surface and on fabric with stainless-steel (SS) yarn electrodes at different distances, i.e., 3 mm and 6 mm.

The electrochemical activity of liquid PEDOT:PSS is given in Figure 1. It should be noted that measurement with an inert Pt electrode showed no ionic behavior from the electrode that was differently showed by the stainless-steel electrode. From the measurement using stainless-steel yarn as the working electrode, it can be seen from the diagram in Figure 1 that two consecutive weak oxidation peaks appeared at 1.05 V and at 1.4 V, while a single and strong reduction peak is observed at 0.8 V. The reduction peak increases with the root mean square of the scan rate, confirming a diffusion controlled process. The results confirmed one of properties of PEDOT:PSS, which acted as a polyelectrolyte for this electrochemical system. In this redox reaction, diffusion of reactants, which occurred most probably due to the charge transport originated from free charge carriers of the PEDOT in oxidized state, went slower than the formation of the reaction product from its activated complex. Based on the literature, the PSS does not contribute to charge transport directly, but it acts as a template to keep PEDOT in the dispersed state and provide film-forming properties [23].

The samples on the glass surface with SS (stainless-steel) yarn electrodes spaced at two different distances (3 mm and 6 mm) are given in Figure 2. As shown by the cyclic voltammograms (Figure 2a,b), the distance of the electrodes (*d*) had a major influence on the conductivity and capacitive behavior of the samples. The square structure observed between 0–1.8 V regions from the sample with a 3-mm electrode distance provides a visual confirmation of the capacitive behavior (Figure 2a) of the cell, while the higher current intensities define its better conductivity. On the other hand, the sample with a 6-mm electrode distance (Figure 2b) has lower conductivity and weaker capacitive behavior. Similarly, the sample on the fabric surface showed the same behavior (Figure 3). The capacitance of each device can be determined by calculating the surface area integrated between the two curves in the voltammogram. Based on the calculation, the sample on the glass surface has a capacitance of 127 µF and 45 µF for electrode distances of 3 mm and 6 mm respectively, while the sample on the fabric surface showed a capacitance of 66 µF and 36 µF for the 3-mm and 6-mm electrode distance. In both cases, increasing the electrode distance lowered the capacitance. However, it was found that capacitance is higher on the glass surface than on the fabric surface. This was most likely due to the diffusion of ions into the yarn and inner structure of the fabric, which has a strong character of resistance, giving a more discernible result to the lowering of entire capacitive behavior of the device. Additionally, it must be pointed out that the spreading of PEDOT:PSS polymer might have caused an increase in the length of conduction path within the polymer, which eventually reduced the capacitance of the system.

### 2.2. Nyquist and Bode Plots

**Figure 4:**
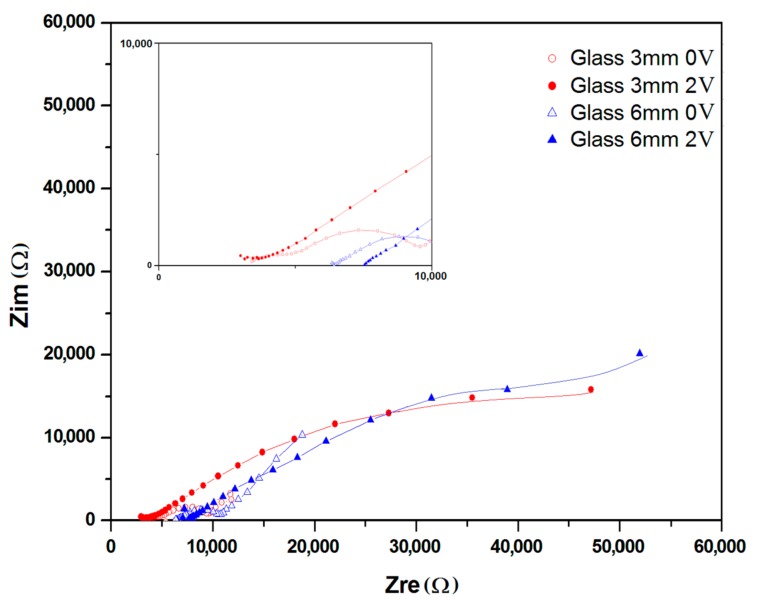
Nyquist plot for samples coated on glass surface at different distance. Inset: high frequency region.

**Figure 5:**
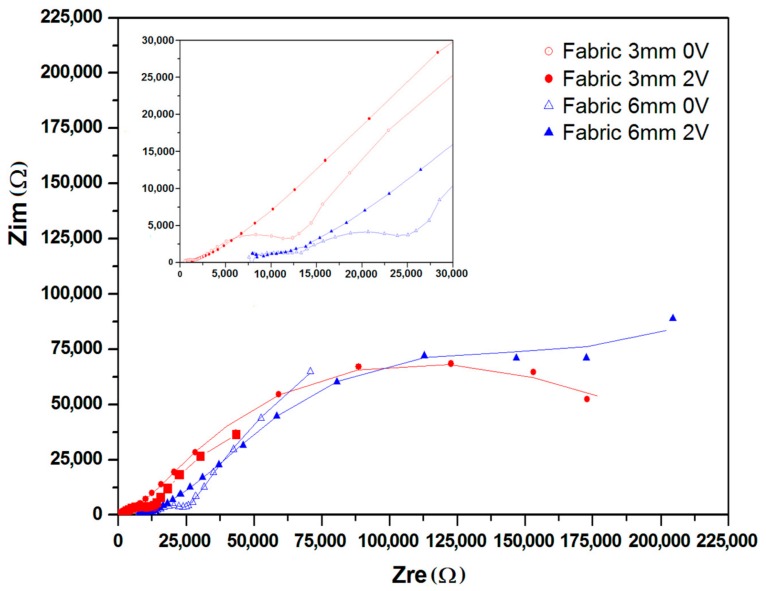
Nyquist plot for samples coated on fabric surface at different distance. Inset: high frequency region.

**Figure 6:**
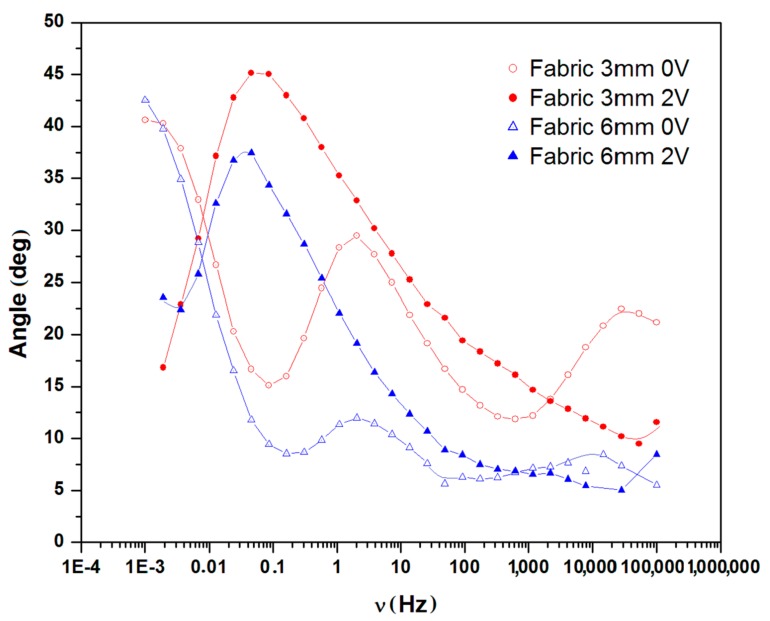
Bode phase plot for samples coated on fabric surface at different distance.

The Nyquist plots of devices made on glass and fabric surfaces each with two different distances between electrodes, i.e., 3 mm and 6 mm are given in Figure 4 and Figure 5 respectively. Each sample was charged at 2 V for a period of 120 min. The plots (insets) exhibit two consecutive semicircles, each of which is followed by a tail formation at the low frequency region, for both glass (Figure 4) and fabric surfaces (Figure 5). The first semicircle with a smaller diameter is related to the bulk PEDOT:PSS polymer, while the second semicircle represents the interface region between PEDOT:PSS and the surface of SS yarn electrodes and the interaction therein. The third section of the plot as defined by a tail-like formation with a phase angle reaching ~45° can be described by the Warburg impedance (Bode phase plot) at samples with 100 kHz–1 mHz frequency region (Figure 6).

The electrochemical impedance analysis (EIS) is a well-known method to determine the conductivity and capacitive behavior of a sample by a single analysis, although it is more common in solution than in solid state. The bulk of solid state materials usually show a single resistive and capacitive value. However, our PEDOT:PSS-based devices exhibited both electronic and ionic characters. From the analysis, the interaction between PEDOT:PSS and the SS electrode surface at the interfacial region was affected by the distance between electrodes, type of electrode and thickness of the PEDOT:PSS layer on the surface of the cell. The obtained impedance spectrums are fitted by an electrical circuit modeling with a frequency response equivalent to that of the measured sample. Various parameters such as resistors (R), capacitors (C), constant phase elements (CPE or Q) and Warburg (W) elements are used as circuit component. While the resistance (R) is defined as the ratio of voltage to current in a DC (Direct Current) circuit, the impedance (Z) is defined as the ratio of voltage to current in an AC (Alternating Current) circuit. The effect of the current applied at different frequencies is analyzed under voltage. Whereas the real part corresponds to the resistance, the imaginary part refers for the phase shift [24]. For resistive behavior, the impedance (Z) acts like a resistance R, while at phase shift θ equals to 90°, Z is a pure capacitive behavior. The constant-phase element (Q) is a distributed equivalent circuit element that can be viewed as the nonideality of the capacitors. The finite distribution of the sample in space makes it a nonideal element, but other imperfections such as the roughness of the electrodes may also contribute here. While an ideal capacitance has *n* equal to 1, the rough surface and nonuniform current distribution deviates from 1. On the other hand, the Warburg element (W) defines the diffusion of the ions to the surface with a θ phase shift of 45° and *n* value as 0.5.

As shown by Nyquist and Bode, in the phase plots in Figure 4, Figure 5 and Figure 6, the first semicircles that represent the bulk polymer properties—either on glass and fabric surfaces—show no apparent changes of its ionic conductivity. PEDOT:PSS is a p-doped conducting polymer that can be solution-processed to form transparent and conducting thin films. PSS is used to p-dope PEDOT and also improve its water solubility [23]. The PEDOT:PSS complex consisting of PSS chains to which the PEDOT oligomer firmly attaches itself forms a stacked arrangement with the counter-ions [25]. The EIS results indicate the presence of ionization and migration of the electrolyte PSS units, leaving the PEDOT units—which are known to be the conductive counterpart—naked and thus increasing its solid-state conductivity. The presence of Warburg element (Figure 6) also confirms this process related to the electrode and electrolyte interface. As a result, the resistance decreases, while conductivity increases on every charged sample.

### 2.3. Simulation of Circuit Model

**Figure 7:**
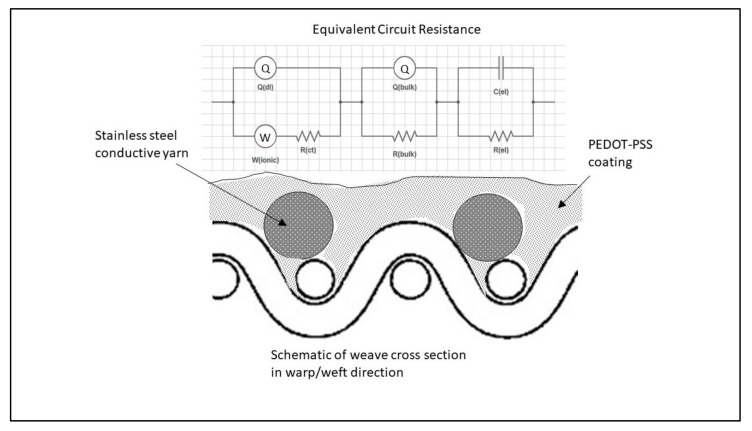
Equivalent circuit models of charged and uncharged device on fabric surface.

The simulated equivalent circuit models with ZSimpWin (version 3.20, Ann Arbor, MI, USA) software compared with experimental results obtained from the Nyquist (Figure 4 and Figure 5) and Bode magnitude plots (R_ct_, C_dl_ and R_el_) are shown in Table 1. Triangles and circles in the curves shown in Figure 4, Figure 5 and Figure 6 represent the points of data from experiments, while the lines show the simulated data taken from the circuit modelling. It can be seen from the graphs that at the lower frequency, the shifted points are more visible, while at the higher frequency, the regions are mostly similar. The results showed two distinct equivalent circuit models of (QR)(QR)(QR) for the uncharged and of (QR)(QR)(Q(RW)) for the charged samples (Figure 7), with χ^2^ defined as the sum of the squares of the residuals minimized to 10^−4^ error. As can be seen in the Table 1, R_ct_ corresponds to the ionic charge transfer resistance, Q_dl_ and R_el_ correspond to the double-layer phase element and electronic resistance at the electrode surface and the solution interface, while Q_bulk_, R_bulk_ and W_ionic_ each correspond to the bulk constant phase element (or capacitance), resistance and Warburg element respectively.

It can be seen from Table 1 that the Q_el_ constant phase element and the R_el_, electronic resistances mostly increased for the charged samples except for the device on fabric with a 3-mm distance, and differs significantly with the electrode distance or the coating on fabric or glass material. The resistance given by extrapolating the end point of the circle related to the electronic conductivity decreased when charged. The second semicircle related to the grain boundaries (R_bulk_) defined by the Brick Layer Model dropped drastically on charged samples, while they altered with the electrode distance. Since the conductivity of a material is an extensive quantity and depends on its dimensions and its electrical resistance, the coating thickness also affects the conductivity. During the course of measurements, we found that samples prepared with thinner PEDOT:PSS coating showed no apparent peak on CV (Cyclic Voltammetry) measurements neither any accurate EIS results, and therefore, cannot be presented in this report. This is due to the absorption of the PEDOT:PSS solution into the fabric structure of the device samples. It is common knowledge that polyester from which the fabric was made is an insulating material and therefore does not allow transport of charges through it, which in other words is of high resistance. For this reason, absorption of polymer is not desirable and must be avoided. From the data in Table 1, it can also be seen that the double-layer capacitance (Q_dl_) and the charge transfer resistance (R_ct_) values decreased for the charged samples on glass surface, while increased for samples prepared on fabric. The presence of the Warburg element for the charged samples proved that the diffusion of ions to the SS yarn electrode surface also affected the value of R_ct_. Therefore, the migration of the PSS counterpart on the fabric surface was more challenging and then decreased the ionic charge transfer.

## 3. Materials and Methods

**Figure 8:**
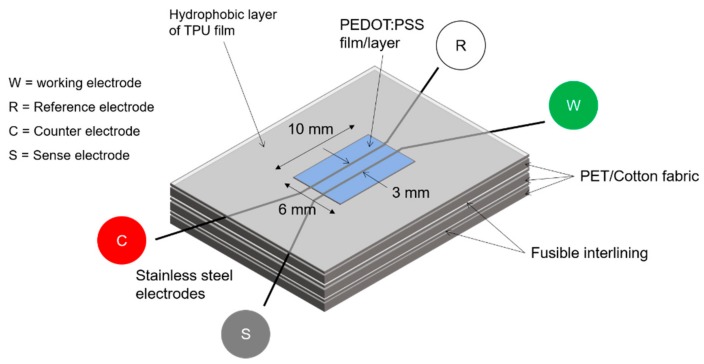
The design of energy storage device used in the experiments and its measurement set-up.

**Device preparation**. Devices used for these experiments were slightly modified from the standard design we had developed in our earlier works [2,6]. Two threads of electrodes were utilized: one strand acted as counter and reference electrode, while the other was functioned as working electrode. Two different distances were set between the two electrodes, i.e., 3 mm and 6 mm. As shown in Figure 8, the cell was prepared on a three-layered of 5 cm × 5 cm twill woven polyester-cotton fabric having a warp and weft density of 42 yarns/cm and 29 yarns/cm respectively. The electrode yarns were stitched on the upper layer of the textile substrate. Two pieces of hot-melt interlining adhesive were used to join each layer together. A thermoplastic polyurethane (TPU) made by Epurex Film Company (Bomlitz, Germany) was applied to mask and provide a hydrophobic effect on the noncell area of the upper surface of the device. Because the electrolyte was of low content (1.5% dispersion in water), seven layers of electrolyte were drop-coated on the cell area of the device surface to provide good coverage. Drying was performed by sequential heating at 90–100 °C for 15 min between each drop.

**Electrodes**. Pure stainless steel Bekaert Bekinox (Bekintex NV, Wetteren, Belgium) was the electrode yarn for the device. An inert platinum (Pt) wire, however, was also used in some devices. The Pt wire (Goodfellow Cambridge Limited, Huntingdon, England) had purity of 99.99% with diameter of 0.40 mm. So, for solid state materials, there were two types of devices: both stainless steels (SS/SS) and both platinum wires (Pt/Pt). Additionally, a device with a combination of SS/Pt electrodes was also prepared to check the movement of ions. For measurement of cyclic voltammetry in a liquid solution system, the inert Pt and stainless-steel yarn were also used and compared as a working electrode, while Ag/AgCl was employed as a reference electrode.

**Electrolyte**. M121 PEDOT:PSS AI 4083 (Ossila Ltd., Sheffield, UK) was used as electrolyte. The electroconductive polymer was a mixture of poly(3,4-ethylenedioxythiophene)-poly(styrenesulphonate) with conductivity of 0.002–0.0002 S/cm. For measurement of cyclic voltammetry in the solution system, the liquid PEDOT:PSS was measured at its dispersion concentration given by the company without any dilution, i.e., 1.5% in water. 0.1 M of NaCl as supporting electrolyte was also included into the dispersion.

**Figure 9:**
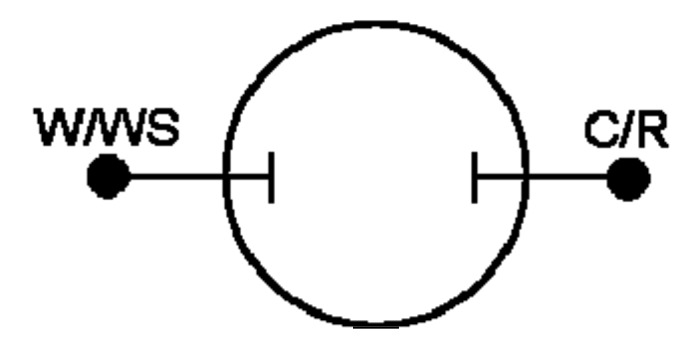
The design of energy storage device used in the experiments and its measurement set-up.

**EIS measurement**. The Advanced Electrochemical System Parstat 2273 of “Princeton Applied Research” was used to perform the EIS (electrochemical impedance spectroscopy) measurements. The instrument was equipped with a built-in software (PowerSuite, Ametek Scientific Instrument, Elancourt, France) for data processing and analysis. The impedance spectra were measured from charged and uncharged devices at the range of frequency of 100 kHz–100 µHz. A two-point probe system was devised using a Princeton Applied Research Parstat 2273 Potentiostat in conjunction with Powersuite^®^ software. Two-electrode experiments measure the whole cell, that is, the sense leads measure the complete voltage dropped by the current across the whole electrochemical cell: working electrode, electrolyte, and counter electrode. The physical setup is shown schematically in Figure 9: two leads, working and reference electrodes, were connected to one side of the sample, with two more leads, the counter and sense electrodes, at the other side of the sample. An equivalent circuit was used to fit the experimental data, with the help of the standalone ZSimpWin v3.20 software.

The charged conditions were achieved via chronoamperometry, where the device was charged at 2 V for 1 h before the EIS reading.

## 4. Conclusions

Electrochemical impedance spectroscopy has been employed in previous research to study the mechanism of our developed textile energy storage device. By presenting the cyclic voltammograms of PEDOT:PSS in the form of electrochemical solution and in its solid state on the glass and the fabric surfaces, ionic behavior of the electroactive polymer PEDOT:PSS has been confirmed through the redox reaction. In general, the distance between electrodes played an important role to the conductivity and capacitive behavior of the PEDOT:PSS. The distribution of PEDOT:PSS polymer film in the solid state also affected its capacitive behavior. The device on a glass surface always exhibited higher capacitive behavior than the device on a fabric surface, which presents a unique challenge that needs to be addressed in later research. From the Nyquist and bode-phase plots of the device, both the ionic and electronic activities of the PEDOT:PSS film in the cell were found, mostly due to the complicated interaction in the interfacial region between the PEDOT:PSS polymer and the stainless-steel electrode. Two different equivalent circuit models were found by simulating the model with the experimental results: (QR)(QR)(QR) for the uncharged samples and (QR)(QR)(Q(RW)) for the charged samples. The results provide a better understanding about the underlying mechanism of the device. This will also lead to a further study on the mechanism and provide more clues about the factors that can be modified to improve the performance of the device.

## Figures and Tables

**Table 1:** Equivalent circuit element for uncharged and charged samples on glass and fabric surfaces.

Sample	Q_el_ * (μS·s^−n^)	n	R_el_ (kΩ)	Q_bulk_ (μF)	n	R_bulk_ (kΩ)	Q_dl_ (μS·s^−n^)	n	R_ct_ (kΩ)	W_ionic_ (μS·s^−1/2^)	X
**On glass**											
3 mm	1.11	0.6479	1.3	5.10	0.7856	2.46	64.6	0.4736	375.48	-	1.25 × 10^−4^
3 mm_ch*)	124.7	0.2144	4.9	4.00	0.9999	1.7	40.23	0.6564	55.43	92.9	1.11 × 10^−3^
6 mm	38.68	0.9052	2.19	0.23	0.4888	17.3	49.43	0.3281	185.1	-	9.90 × 10^−5^
6 mm_ch*)	87.6	0.717	95.7	6.98	0.6834	2.2	22.53	0.002	23.73	9.26	8.02 × 10^−5^
**On fabric**											
3 mm	8.06	0.3754	2.35	902.9	0.7812	128	34.62	0.6781	10.93	-	6.71 × 10^−4^
3 mm_ch*)	293.75	0.0869	1.93	86.5	0.8384	124	56.02	0.3951	171.05	316.6	1.19 × 10^−4^
6 mm	18.0	0.7861	5.41	456.67	0.7329	39.69	38.41	0.7398	18.2	-	4.01 × 10^−4^
6 mm_ch*)	192.8	0.09875	59.9	17.35	0.1392	21.64	32.94	0.4822	215.3	73.7	6.28 × 10^−4^

*) ch = charged.
